# Why Women Engage in Anal Intercourse: Results from a Qualitative Study

**DOI:** 10.1007/s10508-014-0367-2

**Published:** 2014-11-07

**Authors:** Grace L. Reynolds, Dennis G. Fisher, Bridget Rogala

**Affiliations:** 1 Center for Behavioral Research and Services, California State University, 1090 Atlantic Ave., Long Beach, CA 90813 USA; 2 Substance Abuse Foundation Inc. of Long Beach, Long Beach, CA USA

**Keywords:** Heterosexual anal intercourse, Anal sex, Women, Qualitative methods

## Abstract

This study used qualitative methods to assess why women engage in heterosexual anal (receptive) intercourse (AI) with a male partner. Four focus groups which comprised women from diverse ethnicities were conducted. All groups were digitally recorded for transcription; transcripts were analyzed using the methods of grounded theory to determine themes. Women’s reasons for engaging in anal intercourse with a male partner can be described in broad categories including that the women wanted to have anal intercourse, either because of their own desire, to please a male partner, or they were responding to a quid pro quo situation. The riskiness of AI was assessed within relationship contexts. Past experience with AI including emotional and physical reactions was identified. Among the negative physical experiences of AI were pain and disliking the sensation, and uncomfortable side effects, such as bleeding of the rectum. Negative emotional experiences of AI included feelings of shame, disgust, and being offended by something her male partner did, such as spitting on his penis for lubrication. Positive physical experiences included liking the sensation. Many of the women also endorsed positive emotional experiences of AI, including that it was more intimate than vaginal sex, and that it was something they reserved only for special partners. The majority of AI episodes were unplanned and not discussed prior to initiation. Pain during AI was mitigated by the use of lubricants or illicit drugs. Even those women who found pleasure in AI expressed a preference for vaginal intercourse.

## Introduction

Recent interest in heterosexual anal intercourse has been generated from several research perspectives. In the United States, general population surveys have suggested that the prevalence of anal intercourse among heterosexuals has increased over time (Leichliter, 2008). It is not possible to know from these surveys whether the prevalence of anal intercourse is actually increasing, or as some would suggest that the sexual repertoire of Americans has expanded to include anal intercourse, along with oral and vaginal sex (Leichliter, 2008; McBride & Fortenberry, 2010). There may now be less stigma attached to anal intercourse, and respondents to these general population surveys may be more comfortable admitting to the behavior (Mosher, Chandra, & Jones, 2005). Currently in the United States, there are no states that have laws criminalizing anal intercourse (Kelvin, Smith, Mantell, & Stein, 2009). The increase in the reporting of anal intercourse among heterosexuals has implications for public health efforts to educate individuals about the risks of sexually transmitted infections, including those that may be transmitted through anal contact (Fleming & Wasserheit, 1999; Gorbach et al., 2009; Gross et al., 2000; Halperin, 1999; Javanbakht et al., 2010; Tian et al., 2008).

Interest in anal intercourse has also come from research in human immunodeficiency virus (HIV) transmission. Several studies have quantified the increased risk of heterosexual transmission from one act of anal intercourse as compared to one act of vaginal intercourse (Boily et al., 2009; Leynaert, Downs, & de Vincenzi, 1998; Powers, Poole, Pettifor, & Cohen, 2008). The increased risk of HIV transmission through anal intercourse has been well documented in studies of homosexual and bisexual men; however, there has only recently been interest in documenting comparable risks among heterosexual samples. The studies that have used heterosexual samples have generally focused on parts of the world, such as South Africa, that have not only high rates of anal intercourse among heterosexuals, but also high HIV prevalence in the general population and high numbers of concurrent partners among heterosexuals (Kalichman et al., 2011; Thomas, 2009). Partner concurrency and the higher transmissibility of HIV through anal intercourse also make studying heterosexual anal intercourse compelling in the United States where the prevalence of HIV is high mainly in ethnic minority samples, such as African American and Latina women who have sex with men (McLellan-Lemal et al., 2012; Neblett & Davey-Rothwell, 2011; Reynolds, Fisher, & Napper, 2010). According to the U.S. Centers for Disease Control and Prevention (CDC, 2013), 86 % of HIV cases in women are attributable to heterosexual contact: 65 % of HIV infections in African American women and 17 % of HIV infections in Latina women are attributable to heterosexual contact. Research with women who have male partners recently released from jail or prison has also yielded high rates of anal intercourse (Bland et al., 2012; Swartzendruber, Brown, Sales, Murray, & DiClemente, 2011). Harawa and Adimora (2008) linked high incarceration rates among both men and women in the African American community with HIV through a number of mechanisms, including the role incarceration plays in reducing the number of male sexual partners available to African American women.

There is also research literature on heterosexual anal intercourse among drug-using subsamples, which has found a relationship between anal intercourse and both injection and non-injection drug use (Bogart et al., 2005; Lorvick, Martinez, Gee, & Kral, 2006; Powis, Griffiths, Gossup, & Strang, 1995; Risser, Padget, Wolverton, & Risser, 2009; Strang, Powis, Griffiths, & Gossup, 1994; Zule, Costenbader, Meyer, & Wechsberg, 2007), as well as use of prescription drugs and PD5 inhibitors such as Viagra (Fisher et al., 2006). Mackesy-Amiti et al. (2010) found that among drug-using women, anal sex was more likely to occur during transactional sex (sex for drugs or money) and was not associated with emotional closeness.

While this growing body of literature suggests that anal intercourse among heterosexual women may be more prevalent than previously assumed (particularly among drug-abusing samples of women), there is currently very little information about *why* these women are engaging in anal intercourse. While some have suggested that images of sexual behavior found in popular media may influence both men and women’s sexual behavior (Peterson & Hyde, 2010), the extent to which media images play a role in women’s decisions to engage in anal intercourse (or men’s requests for anal intercourse) is unclear. Similarly, while others have suggested that women’s decisions to engage in anal intercourse may be nested within complex gender relationships that privilege male pleasure and female subjugation (Hekma, 2008; Peterson & Hyde, 2010), the extent to which women reference traditional gender roles (e.g., men are interested in sex as conquests, while women are passive recipients of male advances) and sexual scripts (e.g., shared conventions about gender roles during sexual activity) when deciding to engage in anal intercourse remains unclear (Dworkin, Beckford, & Ehrhardt, 2007; Simon & Gagnon, 1986).

### Theoretical Framework

Social cognitive theory (Bandura, 1986) may help explain women’s decisions to have anal intercourse. Bandura stated that human behavior is learned from watching and interacting with other human beings. Women may learn about anal intercourse through male sex partners, and then they may suggest anal intercourse with new sex partners for a variety of reasons, including a desire to be responsive to his desires or because she has learned to like anal intercourse from the experience with a previous sex partner.

Gender stereotypes provide behavioral norms for a variety of social settings; in sexual situations, men and women may be compelled to follow behavioral expectations (Deaux & Lewis, 1984; Sanchez, Crocker, & Boike, 2005). Research has demonstrated that individuals may rely on these behavioral norms and gender stereotypes when engaging in sex with a new partner (Littleton & Axsom, 2003). Through these traditional gender roles and sexual scripts (e.g., gender and role conventions), women have been taught to prioritize their partners’ needs above their own, and this may be a strong motivator for women engaging in anal intercourse when the male partner desires it.

Gender and power theory, which focuses on the sexual division of labor, sexual division of power, and social norms associated with relationships between men and women, may also inform our understanding of heterosexual anal intercourse (Connell, 1987). Wingood and DiClemente (2000) extended Connell’s theory into public health to include behavioral and biological risk factors as explanations for women’s increased risk for HIV. Their model includes alcohol and drug use and high-risk steady partners who have been linked to anal intercourse. DePadilla, Windle, Wingood, Cooper, and DiClemente (2011) validated Wingood and DiClemente’s model with empirical data demonstrating the relationship between theoretical constructs of gender and power and condom use. Pulerwitz, Amaro, De Jong, Gortmaker, and Rudd (2002) found that the construct of sexual relationship power accounted for variation in the use of condoms for vaginal sex among Latina women, with greater perceived relationship power being associated with more condom use; their findings on the importance of relationship power were replicated in a study of anal intercourse in minority female adolescents, where greater relationship power was associated with the ability to refuse anal intercourse with a male partner (Roye, Tolman, & Snowden, 2013).

The current study sought to examine why heterosexual women engage in anal intercourse. Due to the limited nature of previous research on this topic, we opted for a more exploratory approach aimed at uncovering the broad range of reasons that women had for engaging in anal intercourse. Anal intercourse in this study refers to the penetration of a woman’s anus by her partner’s penis, and not the more general category of sexual behaviors, anal sex, which can include anal-oral contact and digital penetration. To enhance the relevance of this work for both the mental health and public health sectors, we also sought to examine women’s perception of risk related to anal intercourse and women’s emotional and physical experiences during the encounter itself.

## Method

Focus group methods were selected to uncover the wide range of reasons that drug-abusing women may have for engaging in heterosexual anal intercourse. Focus groups are particularly well suited for uncovering a full range of opinions, experiences, or concerns about a topic (Krueger, 1994). Given the limited nature of information on this topic, we thought that the types of generative discussions that take place during focus groups would yield the widest range of experiences, opinions, and insight into women’s reasons for and experiences of engaging in anal intercourse. Focus groups were also preferred by the participating outpatient drug treatment program because participants were familiar with group activities and settings.

### Participants

A total of 32 women participated in four separate focus groups about heterosexual women’s experiences with anal intercourse. All participants were recruited through an outpatient drug treatment program and a community-based HIV and sexually transmitted infections (STI) testing program; the testing program was located at the Center for Behavioral Research and Services (CBRS), an organized research center of the California State University, Long Beach (CSULB). Women were invited to participate in the focus groups if they were at least 18 years of age and acknowledged having had anal intercourse with a man during a previous interview at CBRS and had past experience of illicit drug use. The majority had participated in some form of outpatient drug treatment, but some of the women had never received formal treatment for their drug use. All of the women answered “Yes” to the question “Have you ever in your life had receptive anal sex (your partner’s penis in your butt/anus)” during the initial screening procedures, but only 73 % reported having receptive penile-anal intercourse on the brief questionnaire administered immediately prior to the focus groups. Further questioning revealed that all of the participants had had anal intercourse, but some did not count it as such if the man did not ejaculate or if the woman insisted he withdraw because of pain.

The resulting sample consisted of 32 women from diverse ethnic backgrounds: 31 % were White, 41 % were Black/African American, and 28 % were Latino. The average age of the participants was 37 years (SD = 11.02, range 24–56), and 6 % of the women were currently married.

### Procedure

Women who met the screening criteria described above were invited to participate through a verbal invitation, a flyer, and/or a letter, and were offered $50 cash as an incentive. Each focus group was scheduled on a different day and time to maximize participation, but all focus groups were conducted at both the community-based drug treatment and the HIV/STI testing center from which the women had been recruited. Following the recommendations of Krueger (1994), each focus group consisted of 7–10 participants, and all focus groups were conducted by the first author who has experience with group facilitation and has worked extensively with the population served at both the drug treatment center and CBRS. The focus groups were constituted so that all the women in each group were of the same ethnicity; group 1 was African American, group 2 was Latina, and group 3 was White, but group 4 was mixed with approximately equal proportions of African American and White women.

Upon arrival at the focus group location, participants were first informed about the nature of the study and all associated risks and benefits. Informed consent was a two-stage process: women consented first to participate in the focus group and signed an informed consent form approved by the CSULB Institutional Review Board. The second stage consent process required the women to give separate consent to have the focus group digitally recorded for later transcription and coding. Only women who were willing to consent at both stages, that is, to participate in the focus group and to allow the group to be recorded, participated in the final focus groups. None of the women refused to be audio taped.

Women then answered a brief demographic questionnaire that elicited information on their age, self-reported ethnicity, and the number of biological children, whether they had had oral, vaginal, and anal intercourse at any point in their lifetime, and whether their last sexual encounter was with a man or a woman. The demographic questionnaire was followed by a description of focus group procedures and ground rules. Following the recommendations of Krueger (1994), the focus group protocol consisted of five generally worded questions about heterosexual anal intercourse with male partners, how often it had occurred in their lifetime, the frequency of anal intercourse with their current or most recent sexual partner, the context in which the anal intercourse event took place (type of partner, such as new, casual, and regular), the role of alcohol and illicit substances in facilitating the anal intercourse, and other relevant characteristics of the male partners (known to be bisexual, previous incarceration history) and any other information the women were willing to provide concerning the anal intercourse event itself (e.g., lubricants or enemas used, location such as a motel). Participants were allowed to respond spontaneously to each question and were not required to seek permission to speak or speak in a designated order. Although each participant was not required to answer each question, the facilitator did encourage participation from all women and made efforts to elicit diverging perspectives.

### Data Analysis

The audio files produced by the recording equipment in MP3 format were transcribed verbatim and imported into *Dedoose*, an on-line qualitative analysis program that facilitates coding, sorting, and displaying mixed method data. Specific analysis procedures followed many of the recommendations of Grounded Theory (Glaser, 1998; Miles & Huberman, 1994) and unfolded in several phases. In the first phase, the second author read over the transcripts and noted key ideas in the margins (a step known as marginal coding) (Miles & Huberman, 1994). In the second phase, a constant comparison method was used to group and organize the marginal codes conceptually. This inductive process resulted in a hierarchically organized codebook containing codes and subcodes that emerged from the data itself. In the third phase, *Dedoose* was used to mark excerpts from the transcripts. Excerpts were identified both conceptually (based on the beginning and ending of a distinct idea) and contextually (including all necessary information for accurate interpretation). The codebook was then uploaded to *Dedoose* and used to assign applicable codes to the excerpts. *Dedoose* was used to assess inter-rater reliability utilizing a random selection of one-third of the excerpts created by the second author. In most cases, disagreements involved omissions. This occurred when one person applied a code that was overlooked by the other person. When these omissions were counted as disagreements, the kappa coefficient was .79. When these omissions were left out of the calculations, kappa increased to .93, indicating that there were few outright disagreements in coding. All omissions and discrepancies were then discussed by the coders, and a consensus approach was used to assign final codes. Each of these codes and sample quotes are described in detail below.

## Results

The primary goal of the current study was to uncover a wide range of reasons as to why heterosexual, drug-abusing women engage in anal intercourse. Secondary goals included gaining a deeper understanding of the context of the anal intercourse, women’s perceptions of risk related to anal intercourse, women’s emotional and physical experiences during anal intercourse, and the role of substance abuse in all aspects of the anal intercourse encounter. Results related to each of these research questions are described in more detail below.

### Women’s Reasons for Engaging in Anal Intercourse

**Fig. 1:**
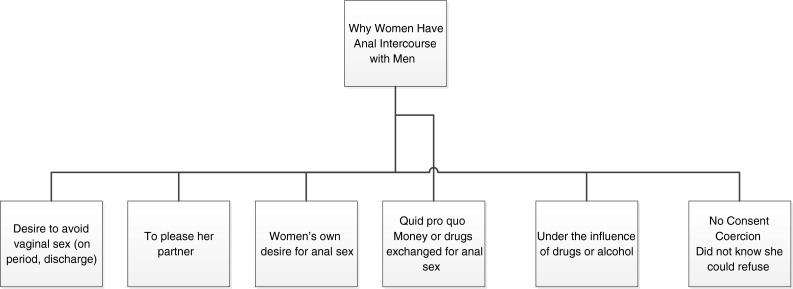
Women’s motivations for having anal intercourse with male partners (*N* = 32)

Results from the current study suggest that heterosexual, drug-using women engage in anal intercourse with male partners for a variety of different reasons. As can be seen in Fig. 1, there were six main reasons that women chose to engage in anal intercourse: they were high and under the influence at the time; because of their own desire; to please a sexual partner; they wanted to avoid vaginal sex (having menstrual period); quid pro quo exchange situations; and situations where they did not explicitly consent, either because they did not know they had a right to refuse or because they were coerced/attacked.

The most frequently reported reason women offered for engaging in anal intercourse was *because they were high* (20/32; 62.5 % of participants).

Every time I have had anal sex it was because I was either extremely drunk or extremely loaded; every time I have had anal sex I was on drugs. (African American, Group 1)

In some of these cases, the women described being more interested in anal intercourse when they were high, suggesting that substance use increased their own sexual desire:

When we do drugs, most drugs we take, we know there’s going to be sex involved… It’s going to be like whether it’s right away (claps hands together), or, like, you know, the minute you do it (claps hands together) –BAM!–your clothes are already off or in the process of getting off. We know what’s coming. Or you made the trip and you go into a motel and you bring all your stuff and you get high and then you are going to have sex. Hours of sex. Hours, hours, hours, yeah. (White, Group 3)

Well most everyone that I know where I came from, homeless, which was under the freeway…everyone is kinky down there, you know. They swear they’re not doing her, her, her, or him, but really she’s doing her and he’s doing him and then it goes back to her type of stuff. Let’s just say the walls are down and nothing is limited…whatever goes, goes. (African American, Group 1)

In other cases, the women described drugs as making them do something they would not ordinarily do, suggesting that they were only willing to engage in anal intercourse when they were high enough to overcome their inhibitions and personal boundaries:

Cocaine makes us do what we would usually not do. Because on the very first date I ever turned, I made $1,700 on Sunset. And this experience I’m talking about, I got paid $75. So, you know, when you start using drugs and shit, it makes you do shit… you have certain boundaries and morals set and it makes you go beneath that. (Latina, Group 2)

Let me tell you, crack will make some people do anything…sell your baby, sell you. Anything! No, crack will make you do anything. (African American, Group 1)

Still others explained that having anal intercourse without using substances would be too painful so they are only willing to have anal intercourse when they are high:

It was the drugs that was the main thing that made me. Because if I wasn’t on drugs, there would be no way in the world first of all I would let a man touch me. You know what I’m saying? If I wasn’t on drugs, you sure nuff not going up my ass. You know what I’m saying? You’re gonna have to break me off right–right, and I gotta be real sprung. You know what I’m saying? (African American, Group 1)

I’m not even sober when I fuck around like that…It was totally not even like worth it when I was drunk. So when I was drunk if I couldn’t take it, I sure the hell can’t take it when I’m sober. (White, Group 3)

Seventeen women (17/32; 53 %) described situations where they did not want the anal intercourse to occur but did not feel she had the right to say no. Thus, while the woman may have granted permission implicitly by not refusing outright, anal intercourse was not something she decided to do in any conscious way.

That’s how low I felt in myself, that it was ok. It got to be where he started doing this on a regular basis. I didn’t feel like I was worth nothing that I allowed him to do it. And I guess because I did not speak up for myself, he really started taking advantage of me. He started doing it to me in my booty–painfully! (African American, Group 1)

You know, it’s something that I do unconsciously. I suffer from depression and certain stuff like that. So a lot of things that I do probably don’t make sense to a lot of people. (White, Group 4)

Fifteen of the participants (15/32; 49 % of participants) described situations where the women engaged in anal intercourse *because they desired it*. In some of these cases, women simply described engaging in anal intercourse because they personally enjoy it:

I do it for enjoyment. (White, Group 4)

I wanted it. I wanted to give it a try. It was done to express our love for one another and I wanted to like do more. I wanted it. I wanted us both to try it. I wanted to do anything I can. I wanted the ultimate workout and he gave it to me. (African American, Group 1)

In other cases, women described only enjoying anal intercourse in specific circumstances such as with people they know and trust very well:

The few times I do it with anybody it has to be with someone I really want to because there are certain things I don’t want to do with certain people. So it’s like a private thing for myself. (Latina, Group 2)

When you’re feeling close to that person…when you are with them longer and know them better…when you are feeling comfortable with them…It is the height of intimacy. Vaginal is just like ok…I think anal is like when you’ve done everything and you finally are…you know the person well. (African American, Group 1)

Others described only having anal intercourse in certain positions or when certain conditions were met:

I won’t have [anal] sex with a man unless I can kick their ass…if I’m gonna give up mine, you’re gonna give up yours. (African American, Group 1)

It has been my choice, you know…like, this is what I want, you know? I only like it in one certain position, so when it does happen, I am in control of how we do it because it is my body, you know? (Latina, Group 2)

Another reason for engaging in anal intercourse occurred in exchange situations (12/32; 37.5 % of participants). For example, some of the women agreed to have anal intercourse in exchange for money:

I tried it a couple of times, but I got paid a very large amount of money. I mean, it had to be about…I got paid about $5,000 the first time I had anal sex. (African American, Group 1)

I met a trucker like that by Skid Row…We were in the back of his cab in his truck and we were getting high and he gave me $200. I was like, oooh, I done come up tonight, you know what I’m saying? I was one of those low-budget hoes, you know, $30 or $40. But for $200, I thought I hit the lotto, right? (African American, Group 1)

Others agreed to have anal intercourse in exchange for drugs:

I was so cracked out in the game that I knew I was going to get a hit when he got through. That’s sad, you know what I’m saying? But that’s how my down was. I knew it wasn’t gonna take too long because my booty tight. It ain’t gonna take long. You know what I’m saying? It hurt! It hurt! It hurt! But all I am thinking about is the hit, the hit, and it ain’t gonna take long. I am going to get a big hit when I get through. And, you know, that’s my experience with anal sex. (African American, Group 1)

I was at that stage in my life where I didn’t care about nothing. And I met this guy. He was a smoker/drug dealer–that’s a smoker that always keeps drugs to sell. And he and I, you know, we lived in this shack, it wasn’t a real house, it didn’t have no electricity, but it was clean and everything. And I became his woman because he had the dope. (African American, Group 1)

Another reason that our sample of drug-abusing, heterosexual women offered for engaging in anal intercourse was *to please a male partner* (9/32; 28 % of participants). In some of these cases, the women agreed to have anal intercourse because the man directly asked (or begged) them to:

It’s always, it’s always ‘baby if you love me, oh baby, let me just have that ass, come on please? (African American, Group 1)

It was me and my husband. He asked, he was curious. We thought about it and then we went on ahead and did it. (African American, Group 4)

In other cases, the women themselves offered to have anal intercourse in an attempt to please their partner:

Like I tried it just to please my dude. I tried it, but I just can’t deal with it. (White, Group 3)

I want to be the type of woman who does satisfy my man in any way…whatever desires he has. (Latina, Group 2)

Seven out of the total sample of 32 women (7/32; 22 % of participants) described situations where the anal intercourse occurred without the woman’s explicit consent. In some of these cases, the man simply initiated anal intercourse:

You ever had the kind that while you were having sex it slipped out…and instead of going boom back in the coochie, they go straight for the ass, knowing that ain’t the coochie? No, no, my coochie way up here! My shit way up here! [Do you think he was deliberately trying to deceive you or was he just confused and in the moment?] He was confused my ass! All the lights were on! Nigga, you see this! I got a hairy coochie! Ain’t no hair around my asshole. He wanted some ass! Yeah, they know what they’re doing. They’re trying to see what you’re gonna say. (African American, Group 1)

We were doing regular sex and then he ask me for my booty. I know he has been to prison, he just got out of prison, and I’m like, nah, don’t do that. And he said, well, let me give you a massage. And I was like, yeah, I got sore legs. Go ahead and give me a massage. He put lotion on my legs and massaged them on up and up my thighs. And he got to the booty and massaged it. Then the next thing you know–BAM!–there he go! He went on and hit it. I was like, no, don’t do that. Then he was real smooth with it. I said, oh, this motherfucker has experience with this thing. (African American, Group 1)

In other cases, the encounter was a violent attack:

No condoms! No lubrication! I am surprised I don’t have AIDS today. You know what I’m saying? He wouldn’t put on no condom. He would flip me and put me in a choke hold, I could hardly breathe, you know? (African American, Group 1)

The very first two times I ever had sex I was raped and I was sodomized. (White, Group 3)

Although the majority of participants said that they did not engage in anal intercourse as a form of birth control, three participants (3/32; 9 %) said that they had engaged in anal intercourse in order to avoid vaginal sex when they were on their period:

I have had anal sex because I was on my period…I just put a tampon in and then yeah. (Latina, Group 2)

### Women’s Perception of Risk

**Fig. 2:**
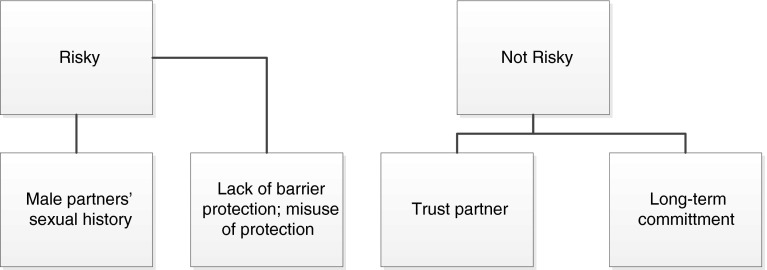
Women’s perceptions of HIV risk associated with anal intercourse (*N* = 32)

The current study also sought to understand women’s perception of risk related to anal intercourse. Results uncovered a variety of factors that were related to women’s perceptions of anal intercourse as risky or not risky (see Fig. 2). Seventeen of the women (17/32; 53 % of participants) described anal intercourse as risky.

From a medical standpoint, I think anal sex is very dangerous because once the tissue breaks, it goes straight to the bloodstream. Also, if you have prolonged anal sex, it ruptures the sphincter, so you’ll be wearing a diaper the rest of your life. (African American, Group 1)

It is risky and I think it is because they be so excited that you have to slow them down…you have to slow them down and let them know, hey, you know, this is a little bit different. It is risky. It is very risky. (African American, Group 4)

Women described two main factors that contributed to their perception of risk: lack of protection (e.g., condoms) and partner’s sexual history. Five of the women (5/32; 16 % of participants) focused on the lack of protection that resulted from not using a condom, using a poor substitute for a condom or using the wrong type of lubricant that could damage the condom:

People sometimes use condoms, but if you use the wrong lotion or lube…you’re gonna break it. (Latina, Group 2)

That’s another thing with using the Saran Wrap or a plastic bag or whatever. It’s like that shit is not gonna protect you like a condom…the thing I’m trying to say is if you are using the wrong contraceptive, that shit is gonna go through that. You feel me? Because it is not proper. That’s not the proper way to perform. (White, Group 3)

Five women (5/32; 15 % of participants) focused on the man’s past sexual history as an indicator of risk, particularly when the man had been to prison or was known to have had sex with other men:

The last one that I was with that I found out had been messing around with other men… I thank God that I never caught anything from him. Cause I was at the most risk of catching HIV ever most in my life with him. (Latina, Group 2)

I know from my personal experience, um, the last person that I was with, um, well, I had anal sex with him. And I had like lots of sex with him. But it was all under the influence. And, um, I’ve heard since then that hedlx is, um, bisexual. So I had an HIV test when I came back here, and, um, was kind of worried about it. But I’m ok. But, um, I put myself at risk with him, cause, uh, I found out that he has had like multiple partners of both sexes. (African American, Group 1)

Six of the women (6/32; 19 % of participants) described an absence of concern about risks associated with anal intercourse, at least at the time the women were engaging in the act and described three main factors that contributed to a lack of concern about risk: being on drugs, trusting their partner, and being in the heat of the moment. Five of the women (5/32; 15.6 % participants) described their substance use as interfering with their concerns about risk or willing to engage in safe sex practices.

I didn’t think twice about not using protection. When I’m using and slamming dope, I don’t care, you know?(White, Group 3)

In my sick head, in my sick addiction, I’m like, oh, fuck it, it is what it is. That’s how I take things, especially with HIV, my brother has it. I take it, like, if I get it, I get it. I’ve injected needles with other people…I’ve done so much shit that like, it’s like a cold to me now. If I get that cold, I get it, and I suffer the consequences. (African American, Group 1)

Four of the women (4/32; 12.5 % of participants) described feeling less at risk because they trusted their partners:

My first time was with my boyfriend who turned out to be my husband. We were dating and my first time was with him. At the time, I didn’t think it was risky. I trusted him. (African American, Group 1)

Like, he is my kid’s dad….It’s just that that was the only man I knew I could come and have sex with instead of going to be a ho-bag at the time. But I mean, I kept on going back to him, running back to him, running back to him. And then, finally, when I realized he had a boyfriend and everybody was telling me the truth, and then he told me, I was like, dude, why didn’t you tell me? You know, like the times we’ve had sex, I’m transferring, getting AIDS. You could have made me aware. (African American, Group 1)

Two of the participants (2/32; 6 % of participants) described how the heat of the moment can interfere with the perception of risk.

Sometimes we don’t think about that when we are in the mood and we are not paying attention to what we are doing. (White, Group 4)

### Women’s Physical and Emotional Experiences During Anal Intercourse

**Fig. 3:**
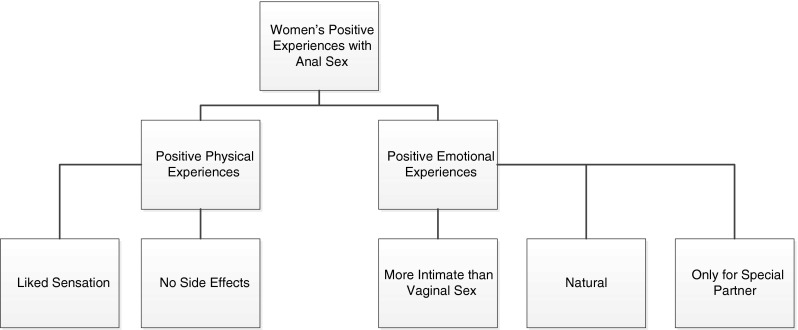
Women’s Positive physical and emotional experiences of anal intercourse (*N* = 32)

The current study also sought to understand women’s physical and emotional experiences during and immediately after anal intercourse. Analysis of women’s descriptions uncovered a variety of contexts that were related to women’s enjoyment or discomfort when engaging in anal intercourse (see Fig. 3). Ten of the women (10/32; 31 % of participants) described anal intercourse as enjoyable.

Personally, I like it; I wanted the ultimate workout and he gave it to me; I do like to have my salad tossed. (African American, Group 1)

Yet, when asked whether they preferred anal intercourse or vaginal intercourse, nearly every woman in the focus groups unanimously expressed a preference for vaginal sex.

Regular! Regular! Regular! We are regulars in here! (Latina, Group 2)

This suggests that most of the women in these focus groups found vaginal intercourse to be more enjoyable than anal intercourse. Nine of the excerpts (9/32; 28 % of participants) specifically discussed the role that substance use played in their experience of pleasure.

I know that anal sex is, uh, I don’t know, for me, it was like I loved it when I was high. (White, Group 3)

Catch me on my come down. On my come down, it was like, I don’t know what it is, I love sex when I’m coming down. When I’m coming down, fuck. I love to fuck on my come down. That is like the best sex ever. I don’t know what it is about it…It’s like you’re half asleep, half awake, like, it’s the best. (Latina, Group 2)

It was also clear that specific contexts or circumstances were typically required in order for the women to enjoy the experience. For example, seven of the women (7/32; 22 % of participants) emphasized the importance of male experience with the use of lubricants for a woman’s enjoyment:

I’m going to put it like this: Hold up, hold up. If you are with a guy who knows what he is doing, it won’t hurt that bad if he takes his time and stuff…it’s really not that bad if the person knows what he is doing. If he is taking his time and stuff and lubricating, then it’s all right. (African American, Group 1)

I mean, to me it was pleasurable. But, like I said, we used a condom and a lubricant. And we took our time, you know? It wasn’t no rush, you know? (African American, Group 4)

Seven of the women (7/32; 22 % of participants) also emphasized the importance of being stimulated in the correct way, or staying relaxed that helped create a more pleasurable experience.

You have to totally, totally, totally relax. If you’re having it, just remember to breathe; I never did have anal sex without, without, like, toys, like clit stimulators or something like that, you know? African American, Group 1)

**Fig. 4:**
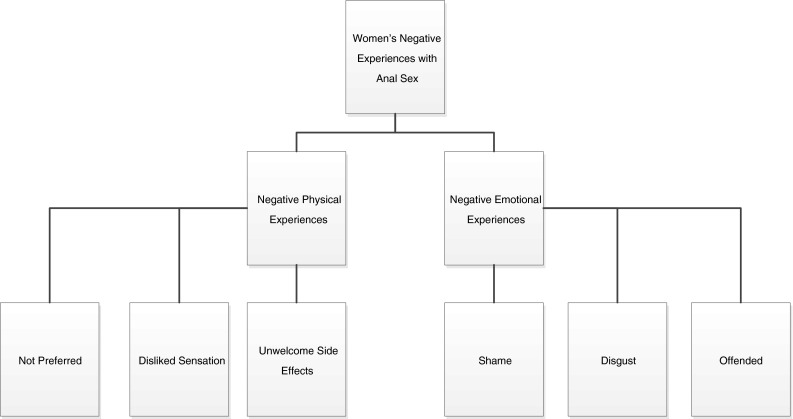
Women’s negative physical and emotional experiences of anal intercourse (*N* = 32)

In contrast to the women who found pleasure in anal intercourse, 15 of the women (15/32; 47 % of participants) focused on the emotional and physical discomfort associated with anal intercourse (see Fig. 4). For many of these women, the experience was physically uncomfortable or downright painful:

No, I don’t even like fingers, don’t even put your finger in there. Party over. Don’t even put your tongue down there…”Baby, do you want your asshole eaten?” No. (African American, Group 1)

I thought that at the time something tore, I don’t know. Yeah, it was very painful. Like, for the first couple of times I tried it, like it felt, seriously it felt like, this is what I thought: I was like, “is my butthole turned inside out?” You know what I mean? It was like it just hurt. It was very, very painful. (African American, Group 1)

Other participants focused on the unwanted side effects.

I hate anal sex, it is very painful. I don’t like the way I have to use the restroom the next day. (African American, Group 1)

My experience was like, as soon as this motherfucker got done fucking me in the ass, I had to go to the toilet. Then, when I took a shit, I wiped my shit and there was blood on the fucking thing. So, yeah, that’s not a good thing to be fucked in the ass. It’s really not. For real, for real, my saying to this day is exit only. You know what I’m saying? Like it’s made for shit to come out not to go in. (African American, Group 1)

Still others felt emotionally humiliated by the experience.

To me, I not only felt sore, but it was demoralizing. It didn’t feel like a normal sexual activity. It felt like I did something wrong. It felt wrong. (African American, Group 1)

When it gets dry, they’ll just pull back out and then they spit and they put it back in… the first time somebody did that, I was like “oh, my.” It was disgusting to me. (White, Group 3)

While some of the women simply expressed discomfort or distaste for anal intercourse, others described specific circumstances that contributed to their dislike of anal intercourse. For example, five of the women (5/32; 15.6 % of participants) described male partners who were so focused on their own pleasure that they failed to consider the women’s experiences:

The anal sex for me is like hard. Because the one time that I did do it, I was drunk and it was fucking shoved in and it hurt. And I was like, it was all bad. (Latina, Group 3)

They don’t know what they are doing. They just want to do it without…they push you all hard instead of going soft…They are focused on themselves and what they want and not, not realizing that it will hurt us more than them. (African American, Group 4)

Others described specific physical deterrents such as condoms, lack of lubrication, or the inability to relax that interfered with women’s ability to experience pleasure:

We started with the rubber, but it seemed like the rubber was irritating me. Even with the lubricant, it was just too much. It kind of traumatized me. (Latina, Group 2)

## Discussion

The current study sought to understand why heterosexual women engage in anal intercourse, their perceptions of risks associated with anal intercourse, and their physical and emotional reactions to anal intercourse. Results from a series of four focus groups with women recruited from a community-based HIV and STI testing program and an outpatient drug treatment program suggested that women had a wide range of views on anal intercourse with a man and motivations for having anal intercourse. Among these motivations were (1) because they were high; (2) the women’s own desire for anal intercourse; (3) a desire to please their partner, (4) in quid pro quo (exchange) situations; (5) because they wanted to avoid vaginal sex; (6) and because they did not consent, either because they did not realize they had to ability to refuse or because they were coerced.

That the majority of women reported that they had anal sex because they were high is not surprising, given the sample of women, which was recruited from a drug treatment and STI testing facility. This current study also found a relationship between anal intercourse, substance use, and *sexual pleasure* among women.

Other reasons noted by the women were that they desired anal intercourse; they wanted to please their partner; they wanted to avoid vaginal sex; the situation was an exchange or quid pro quo one; and situations where the woman did not specifically consent, either because of low self-esteem or coercion. Even in consensual situations, we found that the majority of anal intercourse episodes reported on in this study were initiated by the men, in some cases surprising the women, who either did not expect anal intercourse during the specific encounter or had never done it before. Several women said that the men wanted to have anal intercourse with them in order to initiate them into something they had never experienced before. In their review of heterosexual anal sexuality and anal intercourse behaviors, McBride and Fortenberry (2010) note that the role of the “exotic” in heterosexual anal sexual behaviors and ideas of “gifting” that come from the virginity literature may play a role in anal intercourse and related behaviors between men and women. In our study, several women endorsed the idea that their male partners wanted to facilitate an experience for the women that they had never had before and that anal intercourse was one such new, perhaps exotic experience. Alternatively, women in our study also endorsed the belief that they would only have anal intercourse with special male partners or on special occasions, suggesting that anal intercourse may act as a “gift” from the women to these special partners. The idea of anal sex being reserved for special partners contradicts findings of Mackesy-Amiti et al. (2010) who found that relationship closeness was not associated with anal intercourse in a sample of drug-using women.

Our findings also suggest that a substantial minority of participants never actively consented to having anal intercourse verbally and explicitly. Previous studies on consent for sexual activity may provide some insight into this study’s findings. For example, Hickman and Muehlenhard (1999) reported that most consent for sexual activity was non-verbal and included behavior such as not avoiding the partner’s advances and not explicitly saying “no.” Jozkowski & Satinsky (2013) work, which looked more closely at gender differences in sexual consent, found that women were more likely to consent verbally, and men were more likely to consent nonverbally to sexual activity. The explicit use of verbal consent on the part of women may reflect a traditional conceptualization of women as sexual gatekeepers and provides support for the role of traditional sexual norms influencing heterosexual anal intercourse behavior.

Work by Jozkowski and Peterson (2013) reported that a small minority of college-aged men used deception for both vaginal and anal intercourse. In that study, male college students may have been trying to find a way around women’s likelihood of refusal for sex by proceeding to engage in sexual activity. In many ways, this is a “gray” area between overt sexual consent and sexual coercion, and much of the current literature on sexual assault has not addressed deceptive behaviors within sexual encounters (Jozkowski & Peterson, 2013). Malamuth (1989) noted that some men are willing to engage in aggressive, even coercive sexual behavior, especially if they are unlikely to be caught. The women may have been less likely to overtly refuse the anal intercourse if she was under the influence of drugs. While this was not the case for some of the women in our study who were not shy about saying “no” when anal penetration was painful, many of the women also simply acquiesced. Minieri et al. (2014) noted that experience of intimate partner violence among drug-using women can undermine relationship power. Whatever the truth might be about the “surprise” element involved in the anal intercourse events reported by this sample of women, more study is needed to understand the context of individual risk, consent, and refusal among minority women.

Harawa, Leng, Kim, and Cunningham (2011) reported that more African Americans spend greater parts of their lives single (not married or cohabitating) than do Whites or Latinos, and this is especially true for women. Many social factors have reduced the number of single African American men available to African American women for sexual partnerships, including high rates of incarceration, homicide, and racial disparities in mortality from preventable and chronic health conditions (Adimora & Schoenbach, 2002; Adimora, Schoenbach, & Floris-Moore, 2009; Harawa & Adimora, 2008). Previous research has found that this lack of partners leads to African American women engaging in and accepting condom-less sex, thus lending support for gender and power frameworks to inform our understanding of anal intercourse. Our results indicate that women might consent to anal intercourse because of these same factors. Bland et al. (2012) found that African American men who spent longer than 90 days incarcerated were more likely to report unprotected sex with a woman, including anal intercourse.

A secondary goal of this study was to examine women’s perceptions of risk associated with anal intercourse. Results suggested that a substantial number of the women perceived anal intercourse to be risky after the fact, but a variety of situational factors deterred from their ability to view anal intercourse as risky in the moment, including being in the heat of the moment, trusting their partners, and substance use. Such findings are consistent with previous research (Maynard, Carballo-Dieguez, Ventuneac, Exner, & Mayer, 2009). Factors related to the perception of risk for anal intercourse included partners’ sexual history and a lack of barrier protection during sex. Reynolds, Latimore, and Fisher (2008) reported that sex while high and HIV risk perception were positively associated with anal intercourse in women. Despite some well-publicized scientific studies of the risks of HIV infection from heterosexual anal intercourse, the women interviewed for this study were vague about exactly how their male partners might be placing them at risk. The women acknowledged that gay and bisexual men were a source of HIV infection, and that men who had been to prison and who might have had sex with another man were a source of risk for women. The women did not mention the risks of HIV infection from sex with an injection drug user, though many acknowledged both injection and non-injection drug use by male partners with whom they had had anal intercourse. The women also did not make fine-grained distinctions concerning the male partners’ role in anal intercourse that may have occurred with men. The research literature makes clear distinctions between risks among men who have sex with men from insertive anal intercourse compared to receptive anal intercourse, but the women did not.

Findings from the current study suggest that only a handful of the participants actually enjoyed anal intercourse. Pain as an insurmountable barrier to anal intercourse is consistent with the study by Stulhofer and Adkukovic (2013). Even among the participants who did seem to enjoy anal intercourse, most expressed an explicit preference for vaginal intercourse over anal intercourse and described several specific factors which needed to be in place for them to enjoy the anal intercourse experience. Women who enjoyed anal intercourse specified the need for a partner who was experienced in the use of lubricants and who used them to make anal intercourse more pleasurable for the women. Conversely, women with male partners who were more egocentric about their own needs, or lacking experience with lubricant use, or both, during the encounter almost unanimously described the encounter as painful. These findings were consistent with previous research on lubricant use and women’s preferences during sexual activity (Jozkowski, Peterson, Sanders, Dennis, & Reece, 2014) as well as Stulhofer and Ajdukovic’s study suggesting that partners must undergo a learning process in order to make anal intercourse a routine part of sexual relationships.

### Limitations

The current study has limitations worth noting. First, as with many qualitative approaches, the sample size was small. This, and the fact that the majority of participants were ethnic minority women recruited through community-based HIV testing and outpatient drug treatment programs, limits the generalizability of the findings. However, given the statistics on HIV incidence and prevalence in minority women, the sample was also a strength of the study as these are the women who are most at risk for HIV infection from unprotected heterosexual anal intercourse.

Focus groups are well suited to identifying the range and limits of a specific experience. In the current study, we were able to capture a wide range of reasons for engaging in anal intercourse, factors related to the perception of risk, and contexts related to women’s enjoyment of anal intercourse. But it is important to remember the limits of focus group data. While focus groups are very good at uncovering the range of experience, they are not good at uncovering how common any one experience might be. This is because not every person was asked or required to answer every question. A participant’s silence does not necessarily mean that they did not have the experience. Participation was also limited to English-speaking women, and participants were low-income women. Additionally, the women were willing to discuss a stigmatized behavior in a focus group setting. Therefore, this study does not necessarily represent the views of women who may feel uncomfortable discussing anal intercourse in a group setting.

There was also a methodological finding in this study concerning how questions about anal sex and anal intercourse are phrased. A small number of women gave contradictory answers to the screening questions concerning penile-anal penetration and anal intercourse. This suggests that questions must be carefully worded when studying this behavior.

### Conclusion

This study provides insight for understanding how women perceive receptive anal intercourse with male partners and *why* they engage in anal intercourse. Future research should focus on two of the findings from this study. First, how do women decide who the “special” partners are with whom they will have anal intercourse? This has implications for sexual health, and HIV and STI prevention. The women mentioned trust and longer-term partnerships as being associated with less risky anal intercourse, but there were enough instances where rapport established with a new or casual partner was enough for the woman to designate a man as “special.”

Second, more work is needed on the gray area of consent or lack thereof for novel or exotic sexual behaviors that are unplanned and perhaps new experiences. What constitutes consent for a new experience such as anal intercourse, the first time it happens? Or when it is unplanned and not discussed prior to engaging in sexual activity? Whether the most recent experience of anal intercourse is negative or positive may determine whether the woman will engage in anal intercourse in the future, but does not really answer the question as to whether she consented to it the first time. Not all of the anal intercourse episodes reported by the women in this study occurred within the context of sex trading or drug use, suggesting that a more nuanced framework is necessary for understanding how women handle men who may use deception in their sexual encounters or how women handle the introduction of “experimental” or novel acts into a sexual encounter. Given the potential health risks from anal intercourse, further inquiry into this sexual behavior is warranted.
